# Explainable SHAP-XGBoost models for in-hospital mortality after myocardial infarction

**DOI:** 10.1016/j.cvdhj.2023.06.001

**Published:** 2023-06-14

**Authors:** Constantine Tarabanis, Evangelos Kalampokis, Mahmoud Khalil, Carlos L. Alviar, Larry A. Chinitz, Lior Jankelson

**Affiliations:** ∗ Leon H. Charney Division of Cardiology, NYU Langone Health, New York University School of Medicine, New York, New York; † Information Systems Laboratory, University of Macedonia, Thessaloniki, Greece; ‡ Department of Internal Medicine, Lincoln Medical Centre, Bronx New York

**Keywords:** Acute coronary syndrome, Explainable machine learning, Myocardial infarction, In-hospital mortality, SHAP

## Abstract

**Background:**

A lack of explainability in published machine learning (ML) models limits clinicians’ understanding of how predictions are made, in turn undermining uptake of the models into clinical practice.

**Objective:**

The purpose of this study was to develop explainable ML models to predict in-hospital mortality in patients hospitalized for myocardial infarction (MI).

**Methods:**

Adult patients hospitalized for an MI were identified in the National Inpatient Sample between January 1, 2012, and September 30, 2015. The resulting cohort comprised 457,096 patients described by 64 predictor variables relating to demographic/comorbidity characteristics and in-hospital complications. The gradient boosting algorithm eXtreme Gradient Boosting (XGBoost) was used to develop explainable models for in-hospital mortality prediction in the overall cohort and patient subgroups based on MI type and/or sex.

**Results:**

The resulting models exhibited an area under the receiver operating characteristic curve (AUC) ranging from 0.876 to 0.942, specificity 82% to 87%, and sensitivity 75% to 87%. All models exhibited high negative predictive value ≥0.974. The SHapley Additive exPlanation (SHAP) framework was applied to explain the models. The top predictor variables of increasing and decreasing mortality were age and undergoing percutaneous coronary intervention, respectively. Other notable findings included a decreased mortality risk associated with certain patient subpopulations with hyperlipidemia and a comparatively greater risk of death among women below age 55 years.

**Conclusion:**

The literature lacks explainable ML models predicting in-hospital mortality after an MI. In a national registry, explainable ML models performed best in ruling out in-hospital death post-MI, and their explanation illustrated their potential for guiding hypothesis generation and future study design.

**Key Findings**

• Extreme gradient boosting–based models for the prediction of in-hospital mortality after myocardial infarction (MI) outperformed previously published models and maintained predictive accuracy in previously undescribed subpopulations.
• All models performed best for ruling out in-hospital death post-MI with a high negative predictive value.
• The models’ explainability provided insights into the relationship between post-MI in-hospital mortality with age, sex, and hyperlipidemia.

## Introduction

Factors associated with mortality following a myocardial infarction (MI) have been studied in the past. The Thrombolysis in Myocardial Infarction (TIMI) risk score defined mortality-predicting variables at presentation separately in patients with unstable angina/non–ST-elevation myocardial infarction (NSTEMI)^1^ and in fibrinolytic-eligible patients with ST-elevation myocardial infarction (STEMI).^2^ Specifically for in-hospital mortality post-MI, the Global Registry of Acute Coronary Events (GRACE) score specified 8 risk factors.^3^ These risk scores as well as those of subsequent validating studies were derived from conventional statistical models, such as logistic regressions.^4^

A few examples of machine learning (ML) models for prediction of post-MI in-hospital mortality have recently been reported in the literature.^5^, ^6^, ^7^, ^8^, ^9^, ^10^ Compared to their logistic regression counterparts, these ML models have yielded comparable^6^ or greater^7^ predictive accuracy. However, their analysis is limited to certain patient subpopulations^5^^,^^8^ or to comparison of the models’ area under the receiver operator characteristic curve (AUC).^6^^,^^7^^,^^9^^,^^10^ The literature to date lacks a comprehensive and explainable ML model for the prediction of in-hospital mortality following an MI.

Models lacking explainability can hide potential biases, including racially biased datasets^11^ and missed confounding variables.^12^ A lack of explainability also limits our mechanistic understanding of how a prediction was made. The ensuing inability to justify clinical decisions derived from such ML models has undermined their uptake into clinical practice.^11^ Indeed, studies have established that clinicians view explainability instead of predictive accuracy alone as the limiting step to incorporating ML model outputs into their practices.^13^^,^^14^

The present study generates explainable ML prediction models for in-hospital mortality after an MI, including subgroups defined by MI type and/or sex. We aim to illustrate how explainable ML models can guide both hypothesis generation and the design of subsequent statistical studies, as well as expand population-wide predictions to specific patient subpopulations. To that end, we developed SHAP-XGBoost models because they are explainable and previously exhibited the highest AUC on this topic.^6^^,^^10^

## Methods

### Data source

Data were obtained from the National Inpatient Sample (NIS), a publicly available de-identified database of hospital inpatient stays in the United States, sponsored by the 10.13039/100000133Agency for Healthcare Research and Quality as part of the Healthcare Cost and Utilization Project.^15^ International Classification of Diseases-Ninth Edition-Clinical Modification (ICD-9-CM) codes were used to identify all patients aged 18 years or older with a primary diagnosis of MI between January 1, 2012, and September 30, 2015 (Supplemental Table 1). Baseline characteristics of the study population were obtained using either Elixhauser comorbidities^16^ or the corresponding ICD-9-CM codes (Supplemental Tables 1 and 2). Importantly, the summary statistics in Supplemental Table 2 apply only to the dataset used to train the ML models and do not represent national trends in MI patient characteristics.^17^

### Data preprocessing

The resulting dataset comprised 457,096 records described using 65 variables, namely, 64 predictors and the response variable, in-hospital death. The response variable was imbalanced, containing 434,355 “0” (alive) and 22,741 “1” (deceased) values, resulting in a 19.1 imbalance ratio (Supplemental Table 2). In turn, this dataset of patients aged 18 years or older with a primary diagnosis of MI was divided into 8 subgroups (Table 1).

**Table 1:** Number of patients, testing set AUC scores, sensitivity, specificity, positive predictive value, negative predictive value, and F1 scores corresponding to the overall dataset of all MIs as well as 8 dataset subgroups based on MI type (NSTEMI vs STEMI), sex (male vs female), and their in-between combinations

`Datasets	No. of patients	Testing set					
		AUC (95% CI)	Sensitivity	Specificity	Positive predictive value (Precision)	Negative predictive value	F1 score
All MI cases	457,096	0.922 (0.918–0.926)	0.831	0.848	0.224	0.990	0.352
NSTEMI	322,966	0.903 (0.896–0.910)	0.810	0.824	0.153	0.991	0.257
STEMI	134,130	0.931 (0.925–0.938)	0.864	0.847	0.326	0.986	0.473
Male	281,221	0.932 (0.926–0.937)	0.830	0.868	0.233	0.991	0.364
Female	175,862	0.902 (0.895–0.909)	0.784	0.843	0.231	0.985	0.356
Male with STEMI	91,158	0.942 (0.934–0.949)	0.873	0.868	0.331	0.989	0.480
Female with STEMI	42,965	0.906 (0.895–0.917)	0.818	0.818	0.354	0.974	0.494
Male with NSTEMI	190,063	0.915 (0.907–0.924)	0.816	0.845	0.157	0.992	0.264
Female with NSTEMI	132,897	0.876 (0.865–0.887)	0.752	0.818	0.156	0.987	0.258

AUC = area under the receiver operating characteristic curve; CI = confidence interval; MI = myocardial infarction; NSTEMI = non–ST-elevation myocardial infarction; STEMI = ST-elevation myocardial infarction.

### Creation of predictive models

Each dataset was split into training (70%) and testing (30%) sets. In turn, eXtreme Gradient Boosting (XGBoost) was used to create the predictive model.^18^ XGBoost provides a hyperparameter designed to tune the behavior of the algorithm for imbalanced classification problems. In XGBoost, several parameters need to be selected to maximize model performance. We investigated the combined effect of 6 parameters by evaluating a grid of 3840 combinations using Scikit-learn’s GridSearchCV function (Supplemental Table 3). Because the dataset was imbalanced, stratified folds were created to ensure the same distribution of negative and positive classes was present in each fold. Five-fold stratified cross-validation (CV) was used to finetune the models by splitting the training set into 5 folds to estimate the risk associated with each model. Each model was trained using data from the training folds, and their associated risk was estimated using data from the validation folds.^19^ The CV process was iterated 100 times to decrease both variance and bias, thus creating and evaluating 500 models in each round. The testing set was not part of the training or validation datasets and thus evaluated the model’s performance on previously unseen observations.

### Model explainability

In this study, we used the SHapley Additive exPlanation (SHAP) framework, a local explainability model based on Shapley values.^20^^,^^21^ The Shapley value is the average marginal contribution of a feature value across all possible coalitions. The SHAP framework leverages the internal structure of tree-based models to compute Shapley values in low-order polynomial instead of exponential time, hence reducing computational demands.^20^^,^^22^^,^^23^

### Statistical analysis

Independent samples *t* test and χ^2^ test of independence were used for comparisons between continuous and categorical variables, respectively (Supplemental Table 1). *P* <.05 was considered significant. Statistical analysis was performed using Python’s SciPy library.

## Results

An in-hospital mortality prediction ML model was generated as described in the Methods for the dataset of all MI cases. The demographic characteristics, comorbidities, complications, and hospital characteristics are summarized in Supplemental Table 2. This model was based on 457,096 inpatient admissions and achieved the following performance metrics: AUC 0.922, sensitivity 0.831, and specificity 0.848 (Table 1). In turn, the dataset was divided into 8 subgroups based on MI type (NSTEMI vs STEMI); sex (male vs female); and their in-between combinations (Table 1). Using the same methodology, mortality prediction ML models were generated for each subgroup dataset as well. All subgroup models, except for female patients with NSTEMI (AUC 0.876), achieved AUC >0.9 (Table 1). The model corresponding to male patients with STEMI achieved the highest AUC 0.942, sensitivity 0.873, and specificity 0.868 (Table 1). All models achieved a high negative predictive value ≥0.974 but low positive predictive value ≤0.354 and corresponding F1 scores ≤0.494 (Table 1) in this imbalanced dataset consisting of approximately 5% in-hospital mortality rate.

**Figure 1:**
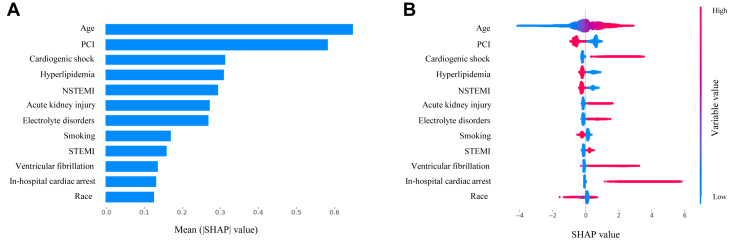
Determinants of in-hospital mortality after myocardial infarction. **A:** SHapley Additive exPlanation (SHAP) variable importance plot ranking the top 12 model variables in decreasing magnitude of contribution (decreasing mean |SHAP value|). **B:** SHAP summary plot, where dots represent distinct patient cases color-coded according to the value of the corresponding variable on the y-axis and their associated Shapley value on the x-axis. Both the magnitude and directionality of contribution to mortality prediction are represented while maintaining the importance ranking order. NSTEMI = non–ST-elevation myocardial infarction; PCI = percutaneous coronary intervention; STEMI = ST-elevation myocardial infarction.

From this point onward, we focused on the ML model corresponding to the overall dataset of all MIs and applied the SHAP framework to explain it. Figure 1A shows a SHAP variable importance plot—a bar chart ranking the top 12 model variables in decreasing magnitude of contribution (mean |SHAP value|) to mortality prediction. The top 3 model variables included age, undergoing percutaneous coronary intervention (PCI), and the development of cardiogenic shock. Figure 1B shows a SHAP summary plot—a beeswarm plot where dots represent distinct cases color-coded according to the value of the corresponding variable on the y-axis and with their associated Shapley value on the x-axis. In this case, we obtain information on both the magnitude and directionality of contribution to mortality prediction depending on the variable’s value, while maintaining the importance ranking order established in Figure 1A. For example, when the binary variable PCI has a value of 1, represented in red, the corresponding patient cases exhibit negative SHAP values (Figure 1B). In other words, patients who underwent PCI following an MI had a lower inpatient mortality.

**Figure 2:**
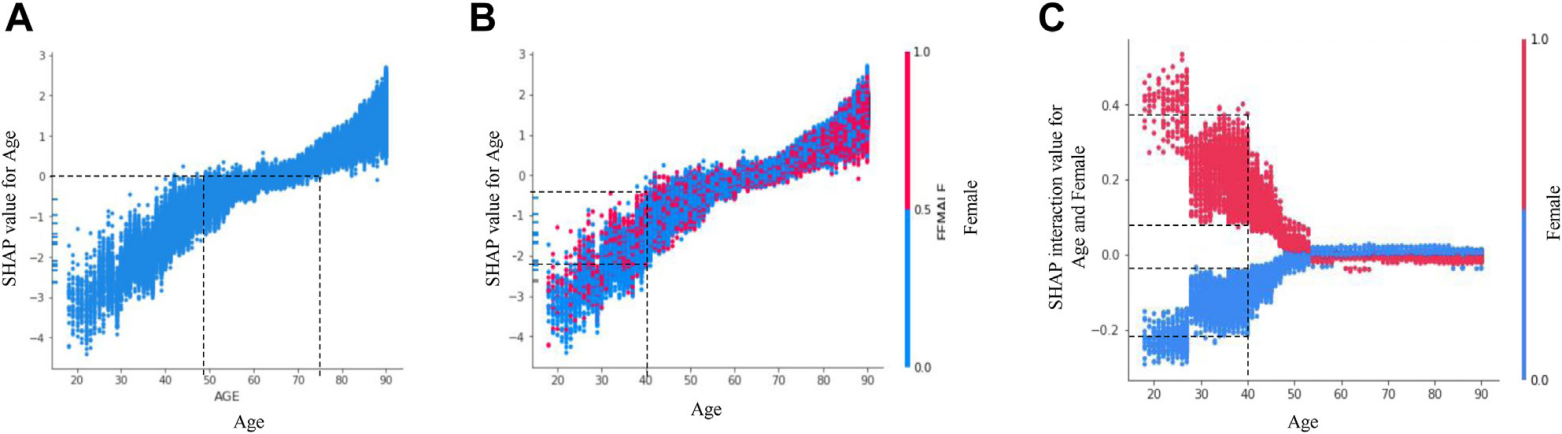
Effect of age and sex on in-hospital mortality after myocardial infarction. **A:** SHapley Additive exPlanation (SHAP) dependence plot showing how increasing age on the x-axis impacts the mortality prediction on the y-axis for every patient case (each dot) in the dataset. **B:** The same SHAP dependence plot color-coded based on the value of an additional variable, in this case sex (red: female; blue: male). **C:** SHAP interaction value dependence plots depicting the interactive effect on mortality prediction between age and sex.

Overall, Figure 1 helps us gain global insights into our ML model. To arrive at local model explanations, other outputs of the SHAP framework focus on individual or pairs of variables. Figure 2A shows a SHAP dependence plot demonstrating how a variable’s value on the x-axis (in this case age) impacts the mortality prediction on the y-axis for every patient case (each dot) in the dataset. In other words, it predicts the changing contribution to mortality prediction with increasing age, notable for a change in directionality from decreasing to increasing mortality between age 50 and 75 years. Figure 2B is the same as 2A, except individual patient cases are color-coded based on the value of an additional variable, in this case sex (red: female; blue: male).

**Figure 3:**
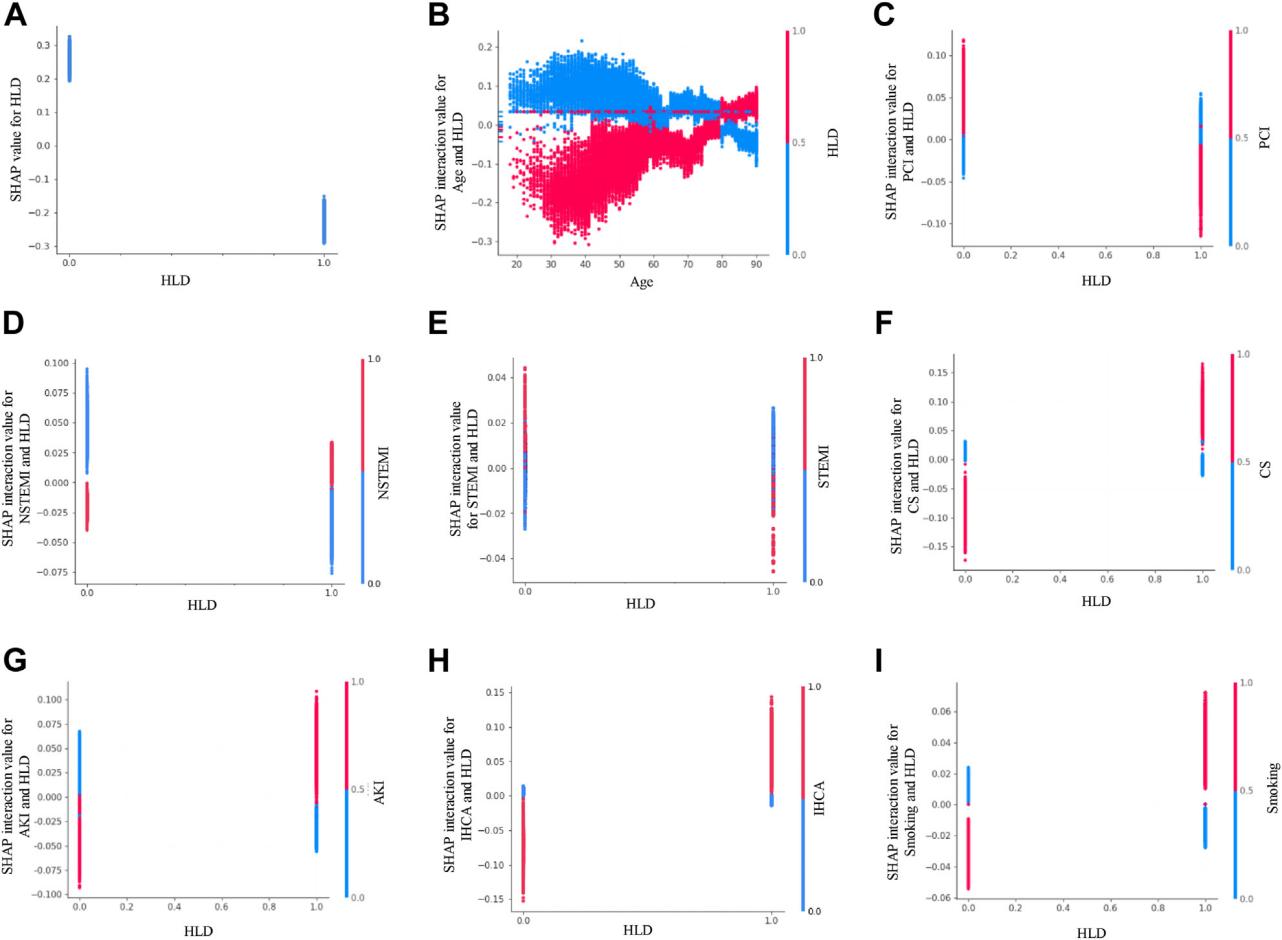
Effect of hyperlipidemia (HLD) on in-hospital mortality after myocardial infarction. **A:** SHAP dependence plot showing how the presence or absence of HLD impacts the mortality prediction on the y-axis for every patient case in the dataset. **B–I:** SHAP interaction value dependence plots depicting the interactive effect on mortality prediction between HLD and age **(B),** percutaneous coronary intervention (PCI) **(C),** NSTEMI **(D),** STEMI **(E),** cardiogenic shock (CS) **(F),** acute kidney injury (AKI) **(G),** in-hospital cardiac arrest (IHCA) **(H),** and smoking **(I).** Abbreviations as in Figure 1.

The cumulative predictive contribution of 2 variables can be decomposed as the additive effect of 4 terms: a constant term; a term for each variable; and a term for the interaction between the 2 variables.^23^^,^^24^ By accounting for the individual effect of each of 2 variables, our model can examine their interaction effect alone. This is represented for age and sex in Figure 2C, which show a SHAP interaction value dependence plot. Similar to Figure 2A, Figure 3A shows a SHAP dependence plot but for the binary variable hyperlipidemia, depicting the magnitude of contribution to mortality prediction depending on its presence or absence. Similar to Figure 2C, Figures 3B–3I show SHAP interaction value dependence plots. The interactive effect on mortality prediction between hyperlipidemia and a variety of variables was iteratively investigated to identify discernible trends, yielding these figures.

## Discussion

All presented ML models exhibited AUC >0.90, except for that corresponding to female patients with NSTEMI (Table 1). To our knowledge, we present the first published ML models for MI in-hospital mortality prediction in the following patient subpopulations: male, female, female patients with NSTEMI, and male patients with STEMI or NSTEMI. Our remaining models for all MIs, STEMI, NSTEMI, and female patients with STEMI (Table 1) outperformed previously published ML models (AUC range 0.80 to 0.91).^6^, ^7^, ^8^, ^9^, ^10^ All presented ML models exhibited a high negative predictive value (≥0.974) and specificity (≥0.818), but comparatively much lower F1 scores and positive predictive value (Table 1). This speaks to the imbalanced (∼5% in-hospital mortality) nature of the dataset rendering the resulting ML models best for ruling out in-hospital death post-MI rather than predicting its occurrence, similar to previous models.^6^^,^^10^

Outside of the traditionally reported metric of accuracy, there is value in this ML approach in its explainability, which is lacking in previous reports.^6^, ^7^, ^8^, ^9^, ^10^ The top-ranked variables by magnitude of contribution to mortality prediction (Figure 1A) included the majority of the GRACE score’s predictor variables,^3^ such as age, cardiac arrest, creatinine level (represented by “AKI”), and ST-segment deviation. The NIS database does not include systolic blood pressure and heart rate values; however, their contribution to mortality can be inferred from the variable “Cardiogenic shock” (Figure 1A). The directional contributions to mortality prediction by variable value (Figure 1B) are also consistent with previous investigations of post-MI in-hospital mortality, which illustrated statistically significant associations with increasing age,^1^, ^2^, ^3^, ^4^ cardiogenic shock,^3^^,^^4^ acute kidney injury,^3^^,^^4^ electrolyte abnormalities (especially potassium),^25^^,^^26^ ST-segment elevation,^3^^,^^4^ in-hospital cardiac arrest,^3^^,^^4^^,^^27^ and ventricular fibrillation.^28^ The counterintuitive association of decreased in-hospital mortality with hyperlipidemia often termed the “lipid paradox”^3^^,^^29^^,^^30^ and with smoking termed the “smoker’s paradox”^31^, ^32^, ^33^ (Figure 1B) has also been previously reported in the literature.

To illustrate the model’s local explainability, we focused on individual and pairwise variable effects (Figures 2A–2C). More specifically, the inflection point in Figure 2A, represented by the change in directionality to mortality prediction (dotted lines), gradually occurs between ages 50 and 75. This is consistent with the TIMI risk score, which included ages 65 and 75 years or older as statistically independent predictor variables for NSTEMI and STEMI mortality, respectively.^1^^,^^2^ It is also important to underline the difference between SHAP values (Figures 2A and 2B) and SHAP interaction values (Figure 2C). For example, 40-year-old female patients who suffered an MI have negative SHAP values (Figure 2B), meaning they exhibit lower in-hospital mortality compared to the overall population. Yet the interactive effect of age and sex illustrates a comparatively increased mortality (positive SHAP interaction values) in female patients of that age group (Figure 2C). Hence, the mortality benefit gained by the individual contribution of age (<50 years) in that subpopulation obscures the underlying mortality increasing effect of female sex, which in turn is revealed by examining the interaction of the 2 variables (Figure 2C). These findings are consistent with previous statistical studies showing a significantly greater post-MI in-hospital mortality in female patients when adjusting for age^34^, ^35^, ^36^ that is observed up to age 60,^37^^,^^38^ in line with SHAP values converging to 0 around age 55 years (Figure 2C).

The previously described counterintuitive association of decreased post-MI in-hospital mortality with hyperlipidemia^3^^,^^29^^,^^30^ persists even after accounting for previous statin use (Figure 1, Figure 3A).^29^ We find that this “lipid paradox” holds in 2 previously undescribed subgroups: <80 years old and patients undergoing PCI (Figures 3B and 3C). The former finding of age dependence suggests that the risk burden of hyperlipidemia needs to accumulate over time to manifest as a measurable increase in early MI mortality. In turn, the paradox does not hold in NSTEMI cases as previously reported,^29^ without a clear pattern emerging in the case of STEMI (Figures 3D and 3E). The paradox does not hold in previously undescribed subgroups, including those developing complications such as cardiogenic shock, acute kidney injury, or cardiac arrest, as well as among smokers (Figures 3F–3I).

Such explainable models are a key first step in addressing clinicians’ understandable hesitancy toward incorporating ML outputs into clinical decision-making.^11^^,^^13^^,^^14^ Future studies should focus on prospectively applying these models in an external population to further characterize their predictive performance.

### Study limitation

A key limitation of the present study is its retrospective design, which precludes any causal inferences. Instead, we view this explainable ML approach as a tool for hypothesis generation, especially given its output’s extensive concordance with previous reports. This approach could also inform the design of future studies by suggesting the need for stratification by certain subgroups.

## Conclusion

The literature to date lacks explainable ML models predicting in-hospital mortality after an MI, which limits its relevance to clinical practice. In a large national registry, explainable ML models performed best in ruling out in-hospital death post-MI, and their explanation provided clinically relevant insights. While recognizing the limitations to model explainability, we sought to define its potential utility in generating hypotheses and informing the design of future investigations.
