# Supplementary material for: Explainable SHAP-XGBoost models for in-hospital mortality after myocardial infarction
Source: Cardiovasc Digit Health J. 2023 Jun 14;4(4):126–32. doi: 10.1016/j.cvdhj.2023.06.001 (PMC10435947; doi:10.1016/j.cvdhj.2023.06.001)
Supplement: Supplementary Materials [file mmc1.docx]

| **Variables** | Code database |
| --- | --- |
| **In-hospital complications** | |
| MI^1,2^ | 4100.x, 4101.x, 4102.x, 4103.x, 4104.x, 4105.x, 4106.x, 4107.x, 4108.x, 4109.x |
| Cardiogenic shock^1,3^ | 78551 |
| Cardiac arrest^4^ | 9960, 9963 |
| Acute kidney injury^3^ | 5845, 5846, 5847, 5848, 5849 |
| Stroke/TIA^5^ | 34660, 34661, 34662, 34663, 430, 431, 4320, 4321, 4329, 43301, 43311, 43321, 43331, 43381, 43401, 43411, 43491, 436, 4358, 4359, 99702 |
| Ventricular fibrillation^4^ | 42741, 42742 |
| Atrial fibrillation^6^ | 42731, 42732 |
| Heart block | 4260, 42610, 42611, 42612, 42613, 4262, 4263, 4264, 42650, 42651, 42652, 42653, 42654, 4266 |
| Papillary muscle rupture^1^ | 4296 |
| Ventricular septal defect^1^ | 42971, 7454 |
| **Baseline health characteristics** | |
| Prior MI^3^ | 412 |
| Prior CABG^3^ | V4581 |
| Prior TIA/stroke^3^ | V1254 |
| Prior PCI^1,7^ | V4582 |
| Heart transplant recipient^7^ | V421 |
| Smoking^1,8^ | 3051, V1582 |
| Dyslipidemia^8^ | 2720, 2721, 2722, 2723, 2724 |
| Known CAD^5^ | 412, 4140, 41400, 41401, 41406, 4142, 4143, 4144, 4148, 4149, V4581, V4582, 41402, 41403, 41407, 44030, 44031, 44032 |
| Carotid artery disease^5^ | 43310 |
| Hemodialysis^1^ | 3995 |
| **Interventions** | |
| PCI^1,8^ | 0066, 3601, 3602, 3605, 3606, 3607 |
| CABG^1,8^ | 3610, 3611, 3612, 3613, 3614, 3615, 3616, 3617, 3619 |

**Supplementary table 1.** International Classification of Diseases, Ninth Edition, Clinical Modification (ICD-9-CM) Codes Used to identify baseline comorbidities and in-hospital Outcomes.

Abbreviations: coronary artery bypass graft (CABG), coronary artery disease (CAD), implantable cardioverter defibrillator (ICD), myocardial infarction (MI), percutaneous coronary intervention (PCI), permanent pacemaker (PPM), transient ischemic attack (TIA).

|  | **Overall**  (N = 457,096) | **Training Set**  (n = 319,967) | **Testing Set**  (n = 137,129) | **p value** | **Alive**  (n = 434,355) | **Deceased**  (n = 22,741) | **p value** |
| --- | --- | --- | --- | --- | --- | --- | --- |
| **Demographic characteristics** |  |  |  |  |  |  |  |
| Age, years (mean, SD) | 67.08 ± 13.87 | 67.09 ± 13.87 | 67.08 ± 13.85 | 0.92 | 66.69 ± 13.81 | 74.69 ± 12.69 | <0.001 |
| Female | 175,862 (38.47) | 123,250 (38.52) | 52,612 (38.37) | 0.33 | 165,724 (38.16) | 10,138 (44.58) | <0.001 |
| Race |  |  |  | 0.638 |  |  | <0.001 |
| White | 324,681 (75.4) | 227,414 (75.45) | 97,267 (75.3) |  | 308,308 (75.33) | 16,373 (76.91) |  |
| Black | 46,136 (10.71) | 32,328 (10.73) | 13,808 (10.69) |  | 44,129 (10.78) | 2,007 (9.43) |  |
| Hispanic | 33,525 (7.79) | 23,347 (7.75) | 10,178 (7.88) |  | 31,915 (7.8) | 1,610 (7.56) |  |
| Asian or Pacific islander | 10,482 (2.43) | 7,312 (2.43) | 3,170 (2.45) |  | 9,931 (2.43) | 551 (2.59) |  |
| Native American | 2,307 (0.54) | 1,627 (0.54) | 680 (0.53) |  | 2,191 (0.54) | 116 (0.54) |  |
| Other | 13,457 (3.13) | 9,388 (3.11) | 4,069 (3.15) |  | 12,826 (3.13) | 631 (2.96) |  |
| Year of hospitalization |  |  |  | 0.739 |  |  | <0.001 |
| **2012** | 121,536 (26.59) | 85,026 (26.57) | 36,510 (26.62) |  | 115,264 (26.54) | 6,272 (27.58) |  |
| **2013** | 120,323 (26.32) | 84,355 (26.36) | 35,968 (26.23) |  | 114,349 (26.33) | 5,974 (26.27) |  |
| **2014** | 121,621 (26.61) | 85,026 (26.57) | 36,595 (26.69) |  | 115,574 (26.61) | 6,047 (26.59) |  |
| **2015** | 93,616 (20.48) | 65,560 (20.49) | 28,056 (20.46) |  | 89,168 (20.53) | 4,448 (19.56) |  |
| Expected primary payer |  |  |  | 0.767 |  |  | <0.001 |
| Medicare | 262,131 (57.45) | 183,580 (57.47) | 78,551 (57.39) |  | 245,152 (56.54) | 16,979 (74.81) |  |
| Medicaid | 34,595 (7.58) | 24,137 (7.56) | 10,458 (7.64) |  | 33,422 (7.71) | 1,173 (5.17) |  |
| Private insurance | 117,733 (25.8) | 82,326 (25.77) | 35,407 (25.87) |  | 114,689 (26.45) | 3,044 (13.41) |  |
| Self-pay | 26,738 (5.86) | 18,791 (5.88) | 7,947 (5.81) |  | 25,841 (5.96) | 897 (3.95) |  |
| No charge | 2,612 (0.57) | 1,835 (0.57) | 777 (0.57) |  | 2,545 (0.59) | 67 (0.3) |  |
| Other | 12,490 (2.74) | 8,765 (2.74) | 3,725 (2.72) |  | 11,953 (2.76) | 537 (2.37) |  |
| Median household income (percentiles) |  |  |  | 0.267 |  |  | 0.122 |
| 0 to 25^th^ | 135,576 (30.32) | 94,924 (30.32) | 40,652 (30.3) |  | 128,667 (30.28) | 6,909 (31.01) |  |
| 26^th^ to 50^th^ | 122,108 (27.3) | 85,633 (27.35) | 36,475 (27.19) |  | 116,061 (27.31) | 6,047 (27.14) |  |
| 51^st^ to 75^th^ | 104,316 (23.33) | 73,088 (23.35) | 31,228 (23.28) |  | 99,210 (23.35) | 5,106 (22.92) |  |
| 76^th^ to 100^th^ | 85,221 (19.06) | 59,432 (18.98) | 25,789 (19.22) |  | 81,001 (19.06) | 4,220 (18.94) |  |
| **Baseline health characteristics** |  |  |  |  |  |  |  |
| Smoker | 194,172 (42.48) | 135,809 (42.44) | 58,363 (42.56) | 0.469 | 187,964 (43.27) | 6,208 (27.3) | <0.001 |
| Hyperlipidaemia | 270,048 (64.18) | 189,184 (64.23) | 80,864 (64.06) | 0.292 | 261,139 (65.3) | 8,909 (42.75) | <0.001 |
| Known CAD | 377,009 (82.48) | 263,908 (82.48) | 113,101 (82.48) | 0.991 | 361,220 (83.16) | 15,789 (69.43) | <0.001 |
| Prior MI | 58,191 (12.73) | 40,629 (12.7) | 17,562 (12.81) | 0.313 | 55,793 (12.85) | 2,398 (10.54) | <0.001 |
| Prior PCI | 71,765 (15.7) | 50,162 (15.68) | 21,603 (15.75) | 0.517 | 69,413 (15.98) | 2,352 (10.34) | <0.001 |
| Prior CABG | 38,126 (8.34) | 26,780 (8.37) | 11,346 (8.27) | 0.286 | 35,990 (8.29) | 2,136 (9.39) | <0.001 |
| Prior TIA/stroke | 30,065 (7.15) | 21,008 (7.13) | 9,057 (7.18) | 0.629 | 28,321 (7.08) | 1,744 (8.37) | <0.001 |
| Atrial fibrillation | 83,637 (18.3) | 58,603 (18.32) | 25,034 (18.26) | 0.636 | 76,953 (17.72) | 6,684 (29.39) | <0.001 |
| Carotid artery disease | 10,658 (2.33) | 7,491 (2.34) | 3,167 (2.31) | 0.522 | 10,161 (2.34) | 497 (2.19) | 0.14 |
| Prior PPM | 13,243 (2.9) | 9,272 (2.9) | 3,971 (2.9) | 0.978 | 12,303 (2.83) | 940 (4.13) | <0.001 |
| Prior ICD | 9,169 (2.01) | 6,420 (2.01) | 2,749 (2.0) | 0.978 | 8,706 (2.0) | 463 (2.04) | 0.759 |
| Prior heart transplant | 41 (0.01) | 28 (0.01) | 13 (0.01) | 0.946 | 32 (0.01) | 9 (0.04) | <0.001 |
| Known VSD | 476 (0.1) | 322 (0.1) | 154 (0.11) | 0.284 | 276 (0.06) | 200 (0.88) | <0.001 |
| On haemodialysis | 14,786 (3.23) | 10,344 (3.23) | 4,442 (3.24) | 0.917 | 13,077 (3.01) | 1,709 (7.52) | <0.001 |
| **Elixhauser comorbidities** |  |  |  |  |  |  |  |
| AIDS | 618 (0.14) | 413 (0.13) | 205 (0.15) | 0.093 | 592 (0.14) | 26 (0.11) | 0.432 |
| Alcohol use disorder | 15,116 (3.31) | 10,530 (3.29) | 4,586 (3.34) | 0.36 | 14,467 (3.33) | 649 (2.85) | <0.001 |
| Anaemia |  |  |  |  |  |  |  |
| Deficiency anaemia | 75,360 (16.49) | 52,830 (16.51) | 22,530 (16.43) | 0.5 | 70,284 (16.18) | 5,076 (22.32) | <0.001 |
| Chronic blood loss anaemia | 3,285 (0.72) | 2,325 (0.73) | 960 (0.7) | 0.339 | 3,079 (0.71) | 206 (0.91) | 0.001 |
| Chronic pulmonary disease | 97,525 (21.34) | 68,283 (21.34) | 29,242 (21.32) | 0.906 | 92,136 (21.21) | 5,389 (23.7) | <0.001 |
| Coagulopathy | 25,378 (5.55) | 17,793 (5.56) | 7,585 (5.53) | 0.694 | 22,495 (5.18) | 2,883 (12.68) | <0.001 |
| Depression | 36,574 (8.0) | 25,641 (8.01) | 10,933 (7.97) | 0.645 | 35,100 (8.08) | 1,474 (6.48) | <0.001 |
| Diabetes with end-organ complications: |  |  |  |  |  |  |  |
| Absent | 141,052 (30.86) | 98,820 (30.88) | 42,232 (30.8) | 0.561 | 134,030 (30.86) | 7,022 (30.88) | 0.953 |
| Present | 33,524 (7.33) | 23,448 (7.33) | 10,076 (7.35) | 0.821 | 31,791 (7.32) | 1,733 (7.62) | 0.092 |
| Drug abuse | 12,399 (2.71) | 8,735 (2.73) | 3,664 (2.67) | 0.273 | 12,057 (2.78) | 342 (1.5) | <0.001 |
| Electrolyte disorders | 103,124 (22.56) | 72,371 (22.62) | 30,753 (22.43) | 0.156 | 91,470 (21.06) | 11,654 (51.25) | <0.001 |
| Heart failure | 3,208 (0.7) | 2,211 (0.69) | 997 (0.73) | 0.187 | 2,662 (0.61) | 546 (2.4) | <0.001 |
| Hypertension | 335,848 (73.47) | 235,186 (73.5) | 100,662 (73.41) | 0.501 | 320,972 (73.9) | 14,876 (65.41) | <0.001 |
| Hypothyroidism | 52,961 (11.59) | 36,992 (11.56) | 15,969 (11.65) | 0.419 | 50,082 (11.53) | 2,879 (12.66) | <0.001 |
| Liver disease | 7,413 (1.62) | 5,162 (1.61) | 2,251 (1.64) | 0.497 | 6,876 (1.58) | 537 (2.36) | <0.001 |
| Malignancy |  |  |  |  |  |  |  |
| Lymphoma | 2,472 (0.54) | 1,741 (0.54) | 731 (0.53) | 0.657 | 2,296 (0.53) | 176 (0.77) | <0.001 |
| Solid tumour without metastasis | 6,553 (1.43) | 4,626 (1.45) | 1,927 (1.41) | 0.297 | 5,989 (1.38) | 564 (2.48) | <0.001 |
| Metastatic cancer | 3,883 (0.85) | 2,687 (0.84) | 1,196 (0.87) | 0.282 | 3,362 (0.77) | 521 (2.29) | <0.001 |
| Neurological disorders | 28,276 (6.19) | 19,923 (6.23) | 8,353 (6.09) | 0.083 | 25,460 (5.86) | 2,816 (12.38) | <0.001 |
| Obesity | 74,558 (16.31) | 52,424 (16.38) | 22,134 (16.14) | 0.042 | 72,124 (16.6) | 2,434 (10.7) | <0.001 |
| Peptic ulcer disease | 109 (0.02) | 84 (0.03) | 25 (0.02) | 0.132 | 105 (0.02) | 4 (0.02) | 0.684 |
| Paralysis | 7,269 (1.59) | 5,064 (1.58) | 2,205 (1.61) | 0.539 | 6,521 (1.5) | 748 (3.29) | <0.001 |
| Peripheral vascular disorders | 56,858 (12.44) | 39,654 (12.39) | 17,204 (12.55) | 0.153 | 52,996 (12.2) | 3,862 (16.98) | <0.001 |
| Psychosis | 12,023 (2.63) | 8,441 (2.64) | 3,582 (2.61) | 0.623 | 11,546 (2.66) | 477 (2.1) | <0.001 |
| Pulmonary circulation disorders | 535 (0.12) | 363 (0.11) | 172 (0.13) | 0.299 | 428 (0.1) | 107 (0.47) | <0.001 |
| Renal failure | 93,992 (20.56) | 65,885 (20.59) | 28,107 (20.5) | 0.472 | 86,688 (19.96) | 7,304 (32.12) | <0.001 |
| Rheumatoid arthritis/  Collagen vascular diseases | 11,486 (2.51) | 8,089 (2.53) | 3,397 (2.48) | 0.319 | 10,888 (2.51) | 598 (2.63) | 0.257 |
| Valvular disease | 973 (0.21) | 670 (0.21) | 303 (0.22) | 0.458 | 796 (0.18) | 177 (0.78) | <0.001 |
| Weight loss | 12,971 (2.84) | 9,074 (2.84) | 3,897 (2.84) | 0.92 | 11,396 (2.62) | 1,575 (6.93) | <0.001 |
| **Acute myocardial infarction type** |  |  |  |  |  |  |  |
| NSTEMI | 322,966 (70.66) | 226,004 (70.63) | 96,962 (70.71) | 0.612 | 311,079 (71.62) | 11,887 (52.27) | <0.001 |
| STEMI | 134,130 (29.34) | 93,963 (29.37) | 40,167 (29.29) | 0.612 | 123,276 (28.38) | 10,854 (47.73) | <0.001 |
| **In-hospital complications** |  |  |  |  |  |  |  |
| Cardiogenic shock | 26,151 (5.72) | 18,376 (5.74) | 7,775 (5.67) | 0.332 | 17,358 (4.0) | 8,793 (38.67) | <0.001 |
| Cardiac arrest | 8,290 (1.81) | 5,796 (1.81) | 2,494 (1.82) | 0.875 | 3,061 (0.7) | 5,229 (22.99) | <0.001 |
| Acute kidney injury | 74,766 (16.36) | 52,297 (16.34) | 22,469 (16.39) | 0.736 | 64,088 (14.75) | 10,678 (46.95) | <0.001 |
| Heart block | 36,800 (8.05) | 25,710 (8.04) | 11,090 (8.09) | 0.557 | 33,784 (7.78) | 3,016 (13.26) | <0.001 |
| Papillary muscle rupture | 126 (0.03) | 94 (0.03) | 32 (0.02) | 0.303 | 79 (0.02) | 47 (0.21) | <0.001 |
| TIA/stroke | 7,540 (1.65) | 5,281 (1.65) | 2,259 (1.65) | 0.949 | 6,343 (1.46) | 1,197 (5.26) | <0.001 |
| Ventricular fibrillation | 13,295 (2.91) | 9,352 (2.92) | 3,943 (2.88) | 0.387 | 9,852 (2.27) | 3,443 (15.14) | <0.001 |
| **In-hospital interventions** |  |  |  |  |  |  |  |
| PCI | 217,871 (47.66) | 152,268 (47.59) | 65,603 (47.84) | 0.119 | 211,406 (48.67) | 6,465 (28.43) | <0.001 |
| CABG | 39,478 (8.64) | 27,819 (8.69) | 11,659 (8.5) | 0.035 | 38,088 (8.77) | 1,390 (6.11) | <0.001 |
| **Hospital characteristics** |  |  |  |  |  |  |  |
| Bed number |  |  |  | 0.901 |  |  | 0.395 |
| Small | 58,883 (12.88) | 41,264 (12.9) | 17,619 (12.85) |  | 56,020 (12.9) | 2,863 (12.59) |  |
| Medium | 126,652 (27.71) | 88,657 (27.71) | 37,995 (27.71) |  | 120,318 (27.7) | 6,334 (27.85) |  |
| Large | 271,561 (59.41) | 190,046 (59.4) | 81,515 (59.44) |  | 258,017 (59.4) | 13,544 (59.56) |  |
| Location/teaching status |  |  |  | 0.045 |  |  | <0.001 |
| Rural | 41,285 (9.03) | 28,794 (9.0) | 12,491 (9.11) |  | 38,972 (8.97) | 2,313 (10.17) |  |
| Urban non-teaching | 158,099 (34.59) | 111,023 (34.7) | 47,076 (34.33) |  | 150,490 (34.65) | 7,609 (33.46) |  |
| Urban teaching | 257,712 (56.38) | 180,150 (56.3) | 77,562 (56.56) |  | 244,893 (56.38) | 12,819 (56.37) |  |
| Hospital region |  |  |  | 0.631 |  |  | <0.001 |
| Northeast | 83,621 (18.29) | 58,453 (18.27) | 25,168 (18.35) |  | 79,257 (18.25) | 4,364 (19.19) |  |
| Midwest | 104,894 (22.95) | 73,347 (22.92) | 31,547 (23.01) |  | 99,963 (23.01) | 4,931 (21.68) |  |
| South | 186,092 (40.71) | 130,290 (40.72) | 55,802 (40.69) |  | 176,846 (40.71) | 9,246 (40.66) |  |
| West | 82,489 (18.05) | 57,877 (18.09) | 24,612 (17.95) |  | 78,289 (18.02) | 4,200 (18.47) |  |

**Supplementary table 2.**

The demographic/baseline health patient characteristics, hospital characteristics and in-hospital complications/interventions for the whole dataset broken down by training/testing sets, as well as alive/deceased patients. Only with the exception of “Age” (mean, SD), all other variables are represented by (n, %).

Abbreviations: acquired immune deficiency syndrome (AIDS), coronary artery bypass graft (CABG), coronary artery disease (CAD), implantable cardioverter defibrillator (ICD), myocardial infarction (MI), percutaneous coronary intervention (PCI), permanent pacemaker (PPM), standard deviation (SD), transient ischemic attack (TIA), ventricular septal defect (VSD).

| **Datasets** | **Parameter tuning** | | | | | |
| --- | --- | --- | --- | --- | --- | --- |
|  | 'learning_ rate' | 'max_ depth' | 'n_ estimators': | 'colsample_ bylevel | 'colsample_ bytree' | ‘subsample' |
| All MI cases | 0.1 | 4 | 400 | 0.6 | 0.6 | 0.8 |
| NSTEMI | 0.1 | 3 | 400 | 0.8 | 0.8 | 0.8 |
| STEMI | 0.1 | 4 | 200 | 0.8 | 0.8 | 0.6 |
| Male | 0.1 | 5 | 200 | 0.6 | 0.8 | 0.8 |
| Female | 0.1 | 3 | 300 | 0.8 | 1 | 0.6 |
| Male with STEMI | 0.1 | 3 | 300 | 0.6 | 0.8 | 0.8 |
| Female with STEMI | 0.1 | 3 | 200 | 0.8 | 0.6 | 0.6 |
| Male with NSTEMI | 0.1 | 4 | 200 | 1 | 0.6 | 0.6 |
| Female with NSTEMI | 0.1 | 4 | 200 | 1 | 0.8 | 0.8 |

**Supplementary table 3.**

Different XGBoost hyperparameter values were tested, selecting combinations that maximized predictive power: ‘n_estimators’: [100, 200, 300, 400, 500, 600], ‘learning_rate’: [0.001, 0.1, 0.2, 0.3], ‘max_depth’: [2, 3, 4, 5, 6], ‘subsample’: [0.4, 0.6, 0.8, 1], ‘colsample_bytree’: [0.4, 0.6, 0.8, 1], and ‘colsample_bylevel’: [0.4, 0.6, 0.8, 1]. The combinations with the maximum AUC output are noted in the table. The code is publicly available here: <https://github.com/kalampokis/nis>.

Abbreviations: Myocardial infarction (MI), non-ST segment elevation myocardial infarction (NSTEMI), ST-segment elevation myocardial infarction (STEMI).
